# RETRACTION: NGF Protects Neuroblastoma Cells Against β‐Amyloid‐Induced Apoptosis via the Nrf2/HO‐1 pathway

**DOI:** 10.1002/2211-5463.13950

**Published:** 2024-12-06

## Abstract

**RETRACTION**: R. Su, W. Su, and Q. Jiao, “NGF Protects Neuroblastoma Cells Against β‐Amyloid‐Induced Apoptosis via the Nrf2/HO‐1 pathway,” *FEBS Open Bio* 9, no. 12 (2019): 2063‐2071, https://doi.org/10.1002/2211‐5463.12742.

The above article, published online on 01 November 2019, in Wiley Online Library (wileyonlinelibrary.com), and its correction (https://doi.org/10.1002/2211‐5463.12782) has been retracted by agreement between the journal Editor‐in‐Chief, Miguel A. De la Rosa; FEBS Press; and John Wiley and Sons Ltd. The retraction has been agreed upon following an investigation into concerns raised by a third party, which revealed inappropriate image duplications between this (Figure 2A, 3A, 5A and 6E) and other articles that were previously published or published in the same or following year. Given the extent of the identified issues, the editors have lost confidence in the data presented and consider the conclusions of this manuscript substantially compromised.

**RETRACTION**: R. Su, W. Su, and Q. Jiao, “NGF Protects Neuroblastoma Cells Against β‐Amyloid‐Induced Apoptosis via the Nrf2/HO‐1 pathway,” *FEBS Open Bio* 9, no. 12 (2019): 2063‐2071, https://doi.org/10.1002/2211‐5463.12742.

The above article, published online on 01 November 2019, in Wiley Online Library (wileyonlinelibrary.com), and its correction (https://doi.org/10.1002/2211‐5463.12782) has been retracted by agreement between the journal Editor‐in‐Chief, Miguel A. De la Rosa; FEBS Press; and John Wiley and Sons Ltd. The retraction has been agreed upon following an investigation into concerns raised by a third party, which revealed inappropriate image duplications between this (Figure 2A, 3A, 5A and 6E) and other articles that were previously published or published in the same or following year. Given the extent of the identified issues, the editors have lost confidence in the data presented and consider the conclusions of this manuscript substantially compromised.

